# Supplementary material for: Mixed-Solvent Polarity-Assisted Phase Transition of Cesium Lead Halide Perovskite Nanocrystals with Improved Stability at Room Temperature
Source: Nanomaterials (Basel). 2019 Oct 30;9(11):1537. doi: 10.3390/nano9111537 (PMC6915538; doi:10.3390/nano9111537)
Supplement: Supplementary file 1 [file nanomaterials-09-01537-s001.pdf]

Support Information

Mixed-Solvent Polarity Assisted Phase Transition of Cesium Lead Halide Perovskite Nanocrystals with Improved Stability at Room Temperature

Rui Yun[a], Li Luo[a*], Jing-qi He[a], Jiaxi Wang[a], Xiaofen Li[a], Weiren Zhao[a], Zhaogang Nie[a], Zhiping Lin[a]

[a] School of Physics and Optoelectronic Engineering, Guangdong University of Technology, Guangzhou 510006, China

[*] Corresponding auther.

E-mail adress: luoli@gdut.edu.cn (L. Luo)

Table S1. The abbreviation of the material in this paper.

Sample	$V_{Ethanol}$: $V_{Toluene}$
a	0
b	0.2
c	0.3
d	0.4
e	0.5
f	0.6
g	0.7

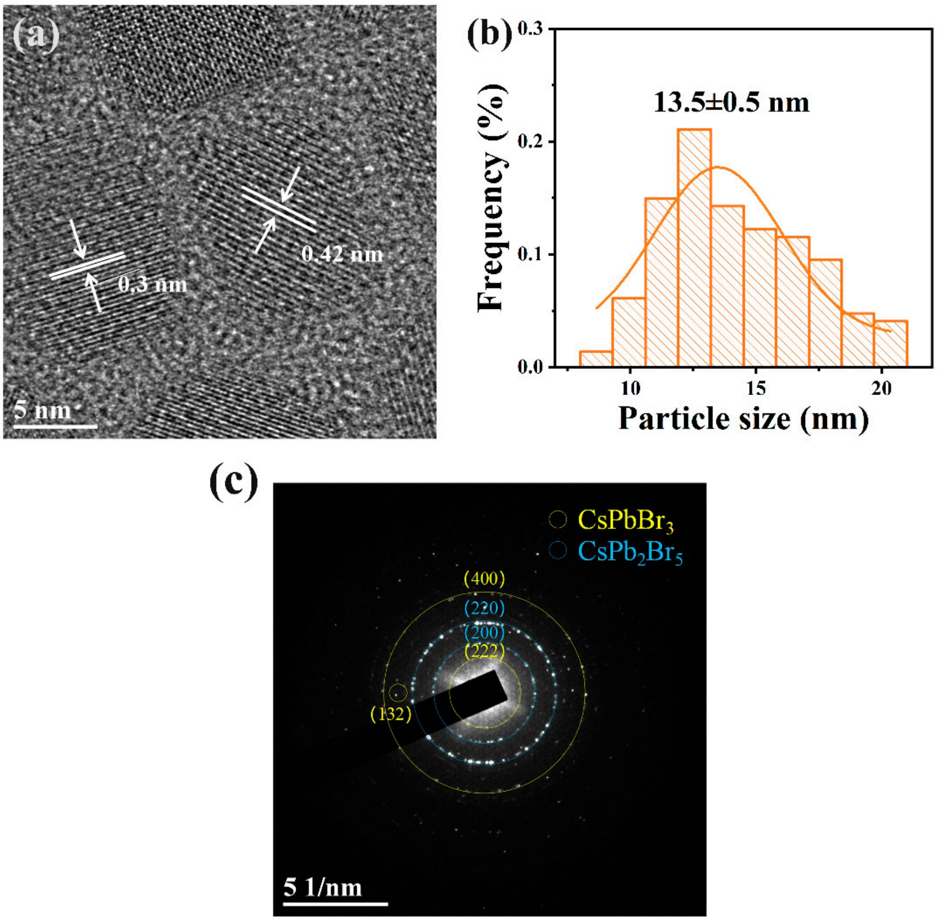

Figure S1. (a) HRTEM image of selected lager sample a; (b); Size distribution analysis of the sample a; (c) The SAED image of sample a.

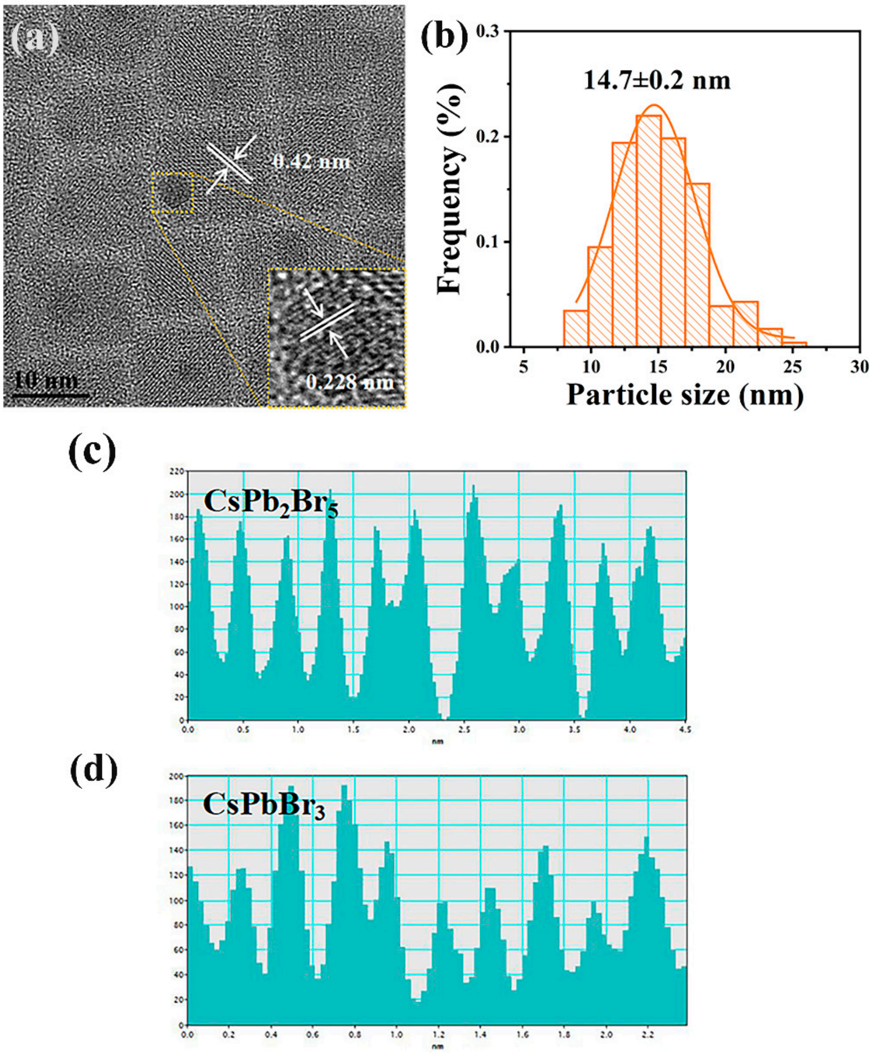

Figure S2. (a) HRTEM image of selected larger sample d; (b) Size distribution analysis of the sample d; Calculation of lattice fringe of (c) $CsPb_2Br_5$ and (d) $CsPbBr_3$.

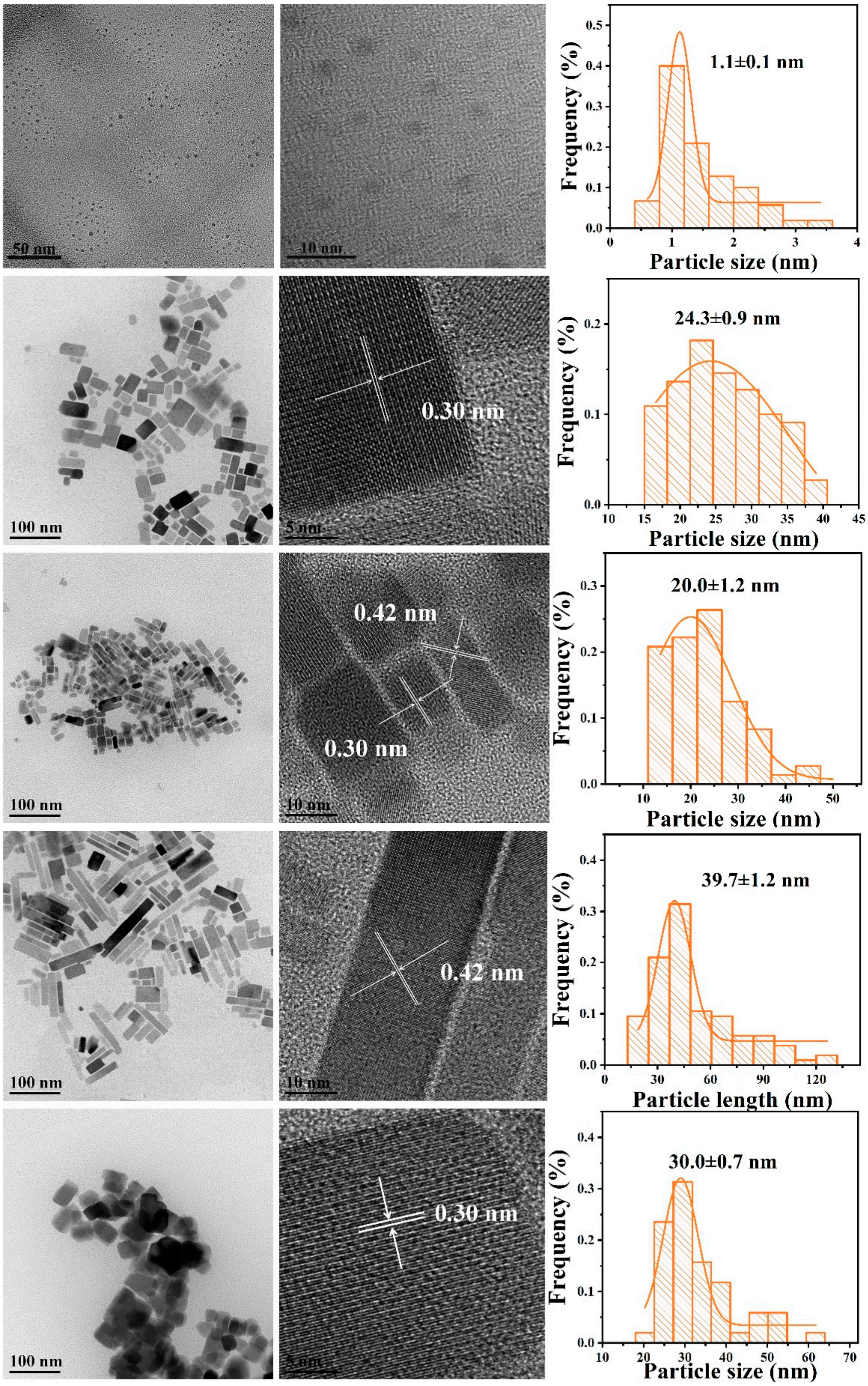

Figure S3. (a, b, c) TEM, HRTEM image and size distribution analysis of sample b; (d, e, f) TEM, HRTEM image and size distribution analysis of sample c; (g, h, i) TEM, HRTEM image and size distribution analysis of sample e. (j, k, l) TEM, HRTEM image and size distribution analysis of sample f. (m, n, o) TEM, HRTEM image and size distribution analysis of sample g.

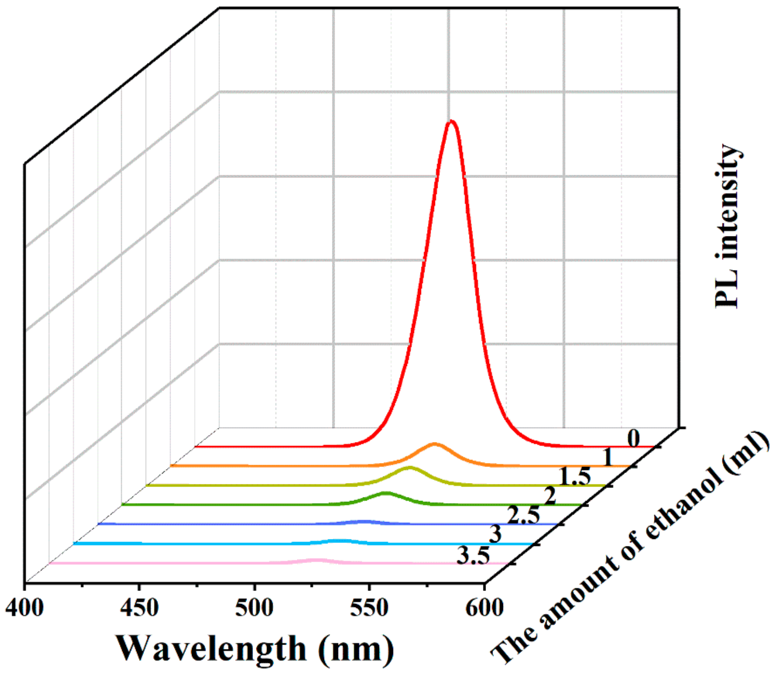

Figure S4. Emission spectra of NCs synthesized with oleic acid and oleylamine under different polar conditions excited by 365 nm.

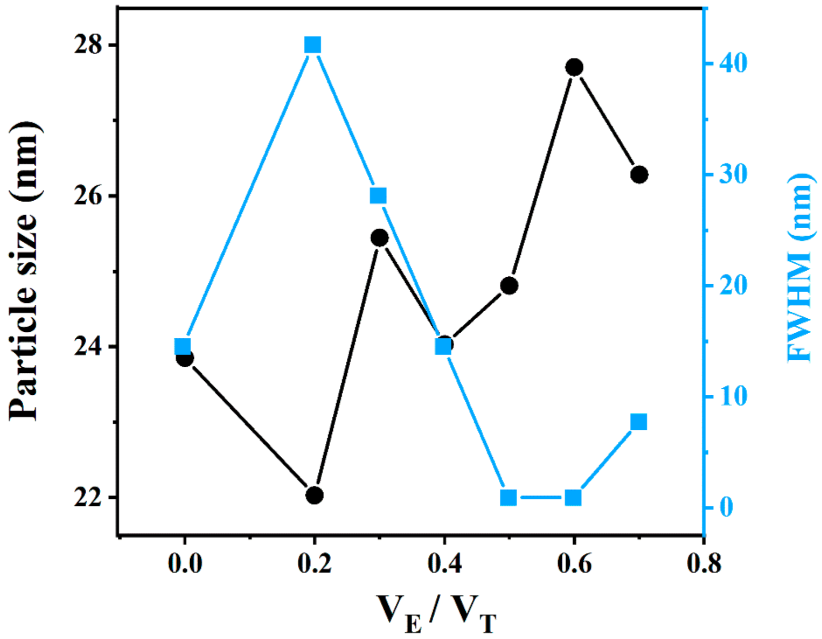

Figure S5. Relationship between V_E/V_T and particle size.

$$A(t) = A_1 \exp(-\frac{t}{t_1}) + A_2 \exp(-\frac{t}{t_2}) + A_3 \exp(-\frac{t}{t_3}) \quad \text{S1}$$

$$\tau_{ave} = \frac{A_1\tau_1^2 + A_2\tau_2^2 + A_3\tau_3^2}{A_1\tau_1 + A_2\tau_2 + A_3\tau_3} \quad \text{S2}$$

Table S2. Exponential fitting results of sample a and d.

Sample	A_1(%)	τ_1 (ns)	A_2(%)	τ_2 (ns)	A_3(%)	τ_3 (ns)	τ_{ave} (ns)
a	40.71	1.3387	47.66	5.0187	11.62	17.4237	9.6765
d	41.58	4.1635	51.49	10.6111	6.93	41.2275	18.2028

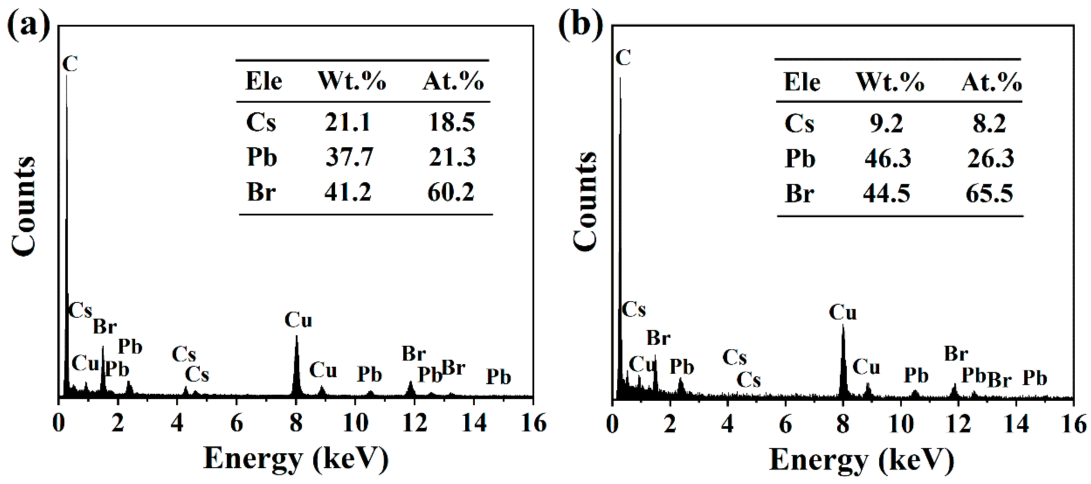

Figure S6. (a) EDS spectra of sample a; (b) EDS spectra of sample d.

Table S3. The ration of Cs to Pb to Br measured by XPS and EDS

Sample	XPS			EDS		
	**Cs**	**Pb**	**Br**	**Cs**	**Pb**	**Br**
a	13.0	16.9	70.2	18.5	21.3	60.2
d	7.52	13.6	78.8	8.2	26.3	65.5

Table S4. PL spectra of NCs synthesized with oleic acid and oleylamine.

**Solvent**	**Ethanol**	**Toluene**
**Polarity**	4.3	2.4
